# Evolving Role of Immunotherapy in Advanced Esophageal Squamous Cell Carcinoma: Are Programmed Death-Ligand 1 (PD-L1) Cutoffs Still Relevant?

**DOI:** 10.7759/cureus.114584

**Published:** 2026-08-16

**Authors:** Praloy Basu

**Affiliations:** 1 Medical Oncology, Netaji Subhas Chandra Bose Cancer Hospital, Kolkata, IND

**Keywords:** combined positive score, esophageal squamous cell carcinoma, immune checkpoint inhibitors, nivolumab, pd-l1, pembrolizumab, tislelizumab, tumor area positivity

## Abstract

Immune checkpoint inhibitors have transformed the management of advanced esophageal squamous cell carcinoma (ESCC) across first-line, second-line, and perioperative settings. Programmed death-ligand 1 (PD-L1) expression has served as the principal biomarker guiding patient selection for these agents, yet it is measured inconsistently across trials and antibody platforms, and its predictive value has come under renewed scrutiny as follow-up data have matured. This review synthesizes the pivotal randomized trials that established anti-programmed cell death protein-1 therapy in ESCC, critically appraises the pooled and patient-level meta-analyses that have re-examined outcomes across biomarker subgroups, and situates recent regulatory reassessment of PD-L1 thresholds within this broader evidence base. Assay heterogeneity between scoring systems, discordance across antibody clones, and the biological distinction between PD-L1 as a prognostic versus a predictive marker are examined as sources of continued uncertainty. The review concludes by considering emerging genomic and microenvironmental biomarkers that may eventually complement or refine PD-L1-based patient selection, and offers a framework for interpreting a single expression threshold as an approximate, assay-dependent stratifier rather than a precise biological boundary.

## Introduction and background

Esophageal cancer remains one of the leading causes of cancer death worldwide, with squamous cell carcinoma accounting for the majority of cases globally, particularly across Asia, Eastern and Southern Africa, and parts of South America [1]. Most patients present with locally advanced or metastatic disease, for which platinum-fluoropyrimidine chemotherapy historically offered a median overall survival (OS) of well under a year. The introduction of programmed cell death protein-1 (PD-1) and programmed death-ligand 1 (PD-L1) pathway inhibitors over the past several years has meaningfully extended survival in esophageal squamous cell carcinoma (ESCC), and has done so in first-line, second-line, and adjuvant contexts. Because these agents are costly and carry immune-related toxicity, PD-L1 immunohistochemistry has been used since the earliest phase 3 trials as a stratification factor and, in several instances, as an explicit eligibility criterion. Yet the story of PD-L1 in ESCC has proven considerably messier than in tumors such as non-small cell lung cancer, owing to differences in scoring algorithms, antibody clones, intratumoral and tumor-stromal heterogeneity, and inconsistent thresholds between agents and regulatory jurisdictions. This review traces how the evidence base evolved from largely PD-L1-agnostic approvals to increasingly biomarker-restricted first-line indications, critically synthesizes the growing body of meta-analytic literature that has re-examined this question at scale, and asks whether a single binary cutoff still serves patients well or whether it obscures a more continuous biological reality.

The clinical stakes of resolving this biomarker question are considerable. ESCC disproportionately affects populations in regions where access to novel systemic therapy is often constrained and where the incremental cost of a checkpoint inhibitor - typically several multiples that of chemotherapy alone - represents a genuine barrier to care rather than a marginal consideration. In health systems where reimbursement is explicitly tied to a PD-L1 threshold, the precise cutoff chosen, and the assay used to measure it, determines not only which patients are eligible for a marginally better outcome but also which patients are exposed to added expense and toxicity without corresponding benefit. A poorly calibrated cutoff therefore has consequences that extend well beyond statistical significance in a forest plot: it shapes real-world formulary decisions, out-of-pocket costs, and the allocation of scarce oncology drug budgets in exactly the regions where disease burden is the highest.

## Review

Biological rationale and the PD-L1 scoring problem

PD-L1 expressed on tumor cells and tumor-infiltrating immune cells engages PD-1 on cytotoxic T lymphocytes, dampening antitumor immunity; blockade of this axis can restore T-cell function against the tumor. In ESCC, PD-L1 is expressed on tumor cells in roughly 40%-60% of cases depending on the assay and cutoff used, and this proportion has driven trial-specific patient stratification. Two scoring systems dominate the ESCC literature: the combined positive score (CPS), which sums PD-L1-staining tumor cells, lymphocytes, and macrophages relative to total viable tumor cells, and the more recently introduced tumor area positivity (TAP) score, a visual-estimation method that scores the proportion of tumor area occupied by PD-L1-positive tumor and immune cells [2]. TAP scoring was developed specifically to reduce the time burden of manual cell counting inherent to CPS - with the original validation study finding that TAP required a median of roughly five minutes per slide compared with approximately 30 minutes for CPS - while achieving high concordance with CPS at clinically relevant cutoffs [2]. Concordance studies comparing TAP and CPS in gastric and esophageal cancer samples from patients treated with tislelizumab reported substantial agreement (Cohen's kappa ≥0.64, overall percent agreement ≥82%), supporting their approximate interchangeability at a threshold of 1%, though agreement was less robust at higher cutoffs [3]. Separately, multicenter comparisons of different PD-L1 antibody clones (22C3, SP263, SP142, E1L3N) in ESCC tissue have shown only moderate inter-assay concordance and meaningful inter-observer variability, with the 22C3 and SP263 clones showing higher sensitivity than SP142 [4,5].

Beyond assay mechanics, a more fundamental biological ambiguity complicates the use of PD-L1 as a single number: expression in the tumor compartment and expression in the surrounding stroma appear to carry distinct, and at times contrasting, prognostic implications. A digital-pathology analysis of 194 surgically resected ESCC specimens using machine-learning-aided H-scores found that high tumoral PD-L1 expression was independently associated with prolonged postoperative survival, whereas high stromal PD-L1 expression correlated with less advanced pathological stage and a more durable response to cytotoxic chemotherapy - two related but mechanistically distinct associations that a single combined score can blur together [6]. This finding underscores a point that is easy to lose in a field organized around a single percentage cutoff: PD-L1 immunohistochemistry was designed, and validated, primarily as a predictive biomarker for response to checkpoint blockade, yet much of the observational literature that shaped clinical intuition about PD-L1 was prognostic in nature, correlating expression with survival in surgically resected, chemotherapy-only, or radiochemotherapy-treated cohorts rather than in randomized comparisons of immunotherapy versus its absence. Conflating a marker's prognostic association (its correlation with outcome regardless of treatment) with its predictive value (its ability to identify who benefits specifically from a given therapy) is a recurring source of confusion when clinicians attempt to extrapolate PD-L1 biology across settings, and it is compounded by tumor and stromal signals that do not always move in the same direction [6]. This assay and biological heterogeneity matter enormously in a field where a single percentage-point difference in scoring can determine whether a patient qualifies for reimbursed first-line immunotherapy.

First-line combination therapy: the pivotal trials

The modern era of first-line immunotherapy in advanced ESCC began with five near-simultaneous phase 3 trials, each pairing an anti-PD-1 antibody with platinum-based chemotherapy (or, in one case, with dual checkpoint blockade) and each using a different PD-L1 assay and cutoff.

CheckMate 648 randomized 970 patients with untreated, unresectable advanced, recurrent, or metastatic ESCC to nivolumab plus chemotherapy, nivolumab plus ipilimumab, or chemotherapy alone, with co-primary endpoints of OS and progression-free survival (PFS) hierarchically tested first in patients with tumor-cell PD-L1 ≥1% and then in the overall population [7]. At a minimum follow-up of 13 months, the median OS with nivolumab plus chemotherapy was 15.4 months versus 9.1 months with chemotherapy alone in the PD-L1 ≥1% subgroup (hazard ratio, or HR, 0.54), and nivolumab plus ipilimumab likewise improved OS in this subgroup [7]. Benefit was also demonstrated, albeit more modestly, in the all-comer population, which supported PD-L1-unrestricted approval in several jurisdictions. Longer term follow-up has continued to show durable separation of the survival curves, with higher landmark survival rates maintained in both experimental arms relative to chemotherapy alone, reinforcing that the initial benefit was not a transient early effect [7].

KEYNOTE-590 randomized 749 patients with previously untreated advanced esophageal or Siewert type 1 gastroesophageal junction cancer (73% ESCC) to pembrolizumab plus chemotherapy or placebo plus chemotherapy [8]. Pembrolizumab-chemotherapy improved OS across the ESCC subgroup (HR 0.72), the PD-L1 CPS ≥10 subgroup (HR 0.62), the ESCC-with-CPS ≥10 subgroup (HR 0.57), and the intention-to-treat population regardless of histology (HR 0.73) [8]. The extended five-year follow-up has since confirmed durable benefit that was concentrated in patients with CPS ≥1, with negligible separation of curves in the CPS <1 subgroup [9].

ESCORT-1st evaluated camrelizumab, a domestically developed Chinese anti-PD-1 antibody, plus paclitaxel-cisplatin chemotherapy against placebo-chemotherapy in 596 Chinese patients with untreated advanced or metastatic ESCC [10]. The trial met its co-primary endpoints, with a median OS of 15.3 months versus 12.0 months favoring camrelizumab in the interim analysis; the final analysis, after roughly two additional years of follow-up, confirmed a sustained OS advantage (15.6 versus 12.6 months; HR 0.70) without evidence of delayed or cumulative toxicity, though the authors likewise concluded that the predictive value of PD-L1 alone appeared limited and called for deeper characterization of the tumor immune microenvironment [11].

ORIENT-15 tested sintilimab plus chemotherapy against placebo-chemotherapy in 659 patients with unresectable, locally advanced, recurrent, or metastatic ESCC, meeting its primary endpoints of OS both in the overall population (HR 0.63) and in patients with PD-L1 CPS ≥10 (HR 0.64) [12].

RATIONALE-306 randomized patients globally across Asia, Europe, Oceania, and North America to tislelizumab plus investigator-chosen chemotherapy versus placebo plus chemotherapy, regardless of baseline PD-L1 status, and demonstrated superior OS with tislelizumab across the intention-to-treat population, with a numerically larger benefit in patients with higher PD-L1 expression [13].

JUPITER-06 added toripalimab to paclitaxel-cisplatin chemotherapy in 514 treatment-naïve Chinese patients with advanced ESCC, again unselected by PD-L1 status, and reported significant PFS (HR 0.58) and OS (HR 0.58) improvements with the addition of toripalimab [14].

Collectively, these five trials - spanning four distinct PD-1 inhibitors, three PD-L1 assay-clone combinations, and both CPS and TAP scoring conventions - established immunotherapy plus chemotherapy as the first-line standard of care for advanced ESCC. Yet they also sowed the seeds of the current controversy: each reported a PD-L1-defined subgroup with a larger relative benefit than the unselected population, but none demonstrated convincing benefit, and some suggested possible harm, in patients with the lowest PD-L1 expression. It is also worth noting that the chemotherapy backbones, dosing schedules, and geographic distribution of enrolled patients differed meaningfully across these trials - with CheckMate 648 using fluorouracil-cisplatin, ESCORT-1st and ORIENT-15 using paclitaxel-cisplatin regimens more common in the East Asian practice, and RATIONALE-306 permitting investigator-chosen chemotherapy - which complicates direct cross-trial comparison of PD-L1 thresholds even before assay differences are considered.

Critical synthesis: what do pooled and patient-level meta-analyses show?

Because no single trial was adequately powered to characterize outcomes within narrow PD-L1 strata, substantial independent meta-analytic literature has emerged to synthesize evidence across trials, and this literature, notably, began raising concerns about low-PD-L1 subgroups well before regulators acted. An early meta-analysis of seven trials comprising 1,733 patients found that checkpoint inhibitor therapy improved the median OS overall (HR 0.75), specifically in the PD-L1-positive population (HR 0.61), while also demonstrating a favorable toxicity profile relative to chemotherapy, but this analysis, conducted before several first-line trials matured, could not finely stratify benefit below a PD-L1 positivity threshold [15]. A subsequent systematic review and meta-analysis focusing on combination regimens confirmed consistent OS, response, and toxicity advantages for checkpoint inhibitor-chemotherapy combinations in both first- and second-line settings [16], and a 2024 Bayesian network meta-analysis of first-line combination regimens found no single anti-PD-1 antibody to be clearly superior to another across trials, reinforcing that the choice among currently available agents in ESCC is driven more by regional availability, cost, and label restrictions than by a demonstrated efficacy hierarchy [17].

A more pointed reckoning came from a 2023 JAMA Oncology meta-analysis that pooled individual trial data from nine first- and second-line studies encompassing 4,752 patients specifically to examine outcomes in low-PD-L1 expression subgroups [18]. This analysis found no significant OS benefit from adding a checkpoint inhibitor to chemotherapy in patients with a tumor proportion score below 1%, while the OS benefit with CPS-based patient selection remained statistically significant, albeit modest, even below a CPS of 10 - a finding that directly foreshadowed, and arguably informed, the FDA's own subsequent pooled analysis and the 2024 Oncologic Drugs Advisory Committee (ODAC) proceedings discussed below [18]. A 2024 updated meta-analysis incorporating 13 trials and more than 6,500 participants likewise confirmed that PD-1/PD-L1 inhibitors combined with chemotherapy improved OS and objective response rate in advanced disease, but, notably, did not significantly improve PFS in aggregate, a discordance between PFS and OS effect sizes that the authors attributed in part to the dilution of benefit within low-PD-L1 subgroups pooled into the overall estimate [19]. Most recently, a 2026 patient-level meta-analysis reconstructing individual survival data from 13 randomized trials (n=6,672) explicitly cautioned against uncritical cross-trial interpretation of PD-L1 thresholds, noting that while the data supported PD-L1 ≥10 as a reasonably robust cutoff for treatment selection and confirmed an absence of OS benefit below a score of 1, the underlying trials used non-interchangeable scoring systems and patient populations, such that any single numeric threshold applied indiscriminately across drugs risks misclassifying patients [20].

Taken together, this meta-analytic literature performs a valuable corrective function relative to any single trial: it converges on the qualitative conclusion that PD-L1-negative or very-low-expressing ESCC derives little to no OS benefit from adding a checkpoint inhibitor to chemotherapy, while also converging on the more cautionary conclusion that the specific numeric cutoff, and the assay used to generate it, cannot be assumed interchangeable across the trials being pooled. In other words, the meta-analyses do not resolve the cutoff question so much as they formalize, at a larger sample size, the same tension that individual trials had already suggested - and they demonstrate that this tension was visible in the peer-reviewed literature roughly one to two years before it became the subject of formal regulatory action.

Second-line monotherapy: establishing PD-1 blockade after progression

Before first-line combinations were validated, anti-PD-1 monotherapy was established in the second-line setting. ATTRACTION-3 randomized 419 patients with advanced ESCC refractory or intolerant to prior fluoropyrimidine-platinum chemotherapy to nivolumab or investigator's choice of taxane, demonstrating superior OS with nivolumab (10.9 versus 8.5 months; HR 0.77) irrespective of PD-L1 expression [21]. Three-year follow-up confirmed a durable survival tail, with three-year OS rates of 15.3% versus 8.7% favoring nivolumab, again without clear PD-L1 dependence [22]. KEYNOTE-181 similarly randomized patients with advanced esophageal cancer after one prior line of therapy to pembrolizumab or chemotherapy; the OS benefit reached significance specifically in patients with PD-L1 CPS ≥10 (12.6 versus 8.4 months in the ESCC-CPS ≥10 subgroup), supporting a PD-L1-selected second-line indication for pembrolizumab distinct from the PD-L1-agnostic nivolumab approval [23,24]. The Chinese ESCORT trial found that camrelizumab improved OS compared to the investigator's choice of chemotherapy as second-line therapy for advanced or metastatic ESCC (8.3 versus 6.2 months) [25]. An indirect comparison of pembrolizumab, nivolumab, and camrelizumab in this second-line population found broadly similar survival benefit across agents, with only modest differences in specific subgroups, underscoring that the choice among available PD-1 inhibitors is influenced more by access and cost than by a clear efficacy hierarchy [26]. Tislelizumab was subsequently validated in the same setting by RATIONALE-302, a global randomized phase 3 study that was, notably, the first to demonstrate significant survival benefit with an anti-PD-1 antibody in an ESCC population spanning both Asia and Europe/North America [27]. A comprehensive 2024 meta-analysis synthesizing this second-line literature alongside the first-line data reaffirmed a consistent OS benefit from checkpoint blockade across lines of therapy, while again flagging the absence of a significant aggregate PFS benefit as an unresolved discordance warranting further biomarker-stratified analysis [19].

Toxicity and the risk side of the risk-benefit equation

The ODAC deliberations, and the broader push to restrict immunotherapy to PD-L1-selected populations, cannot be understood purely as a question of diminishing efficacy at low PD-L1 expression; they are equally a question of unnecessary toxicity exposure when efficacy is absent. Across the first-line combination trials, grade 3-4 treatment-related adverse events were consistently more frequent with the addition of a checkpoint inhibitor to chemotherapy; for example, 49% of patients receiving nivolumab plus chemotherapy in CheckMate 648 experienced grade 3-4 treatment-related adverse events compared with 36% receiving chemotherapy alone, although treatment-related deaths were rare and similar across arms [7]. Immune-related adverse events such as pneumonitis, colitis, hepatitis, and endocrinopathies, while individually uncommon, can be severe, irreversible, and occasionally fatal, and they carry downstream costs in monitoring, corticosteroid use, and quality of life that are not captured by OS or PFS hazard ratios alone. When a PD-1 inhibitor confers no measurable survival advantage in a biomarker-negative subgroup, exposing those patients to this incremental toxicity - and to the substantial additional drug acquisition cost of checkpoint inhibitors - represents a poor risk-benefit trade rather than a neutral one. This asymmetry between a clear toxicity signal and an uncertain or absent efficacy signal in low-PD-L1 expression disease was central to several ODAC members' reasoning and helps explain why the committee reached a near-unanimous vote despite the relatively small number of PD-L1-negative patients available for analysis within any individual trial [28].

Perioperative immunotherapy: adjuvant and neoadjuvant strategies

Immune checkpoint blockade has also moved into curative-intent treatment. CheckMate 577 randomized 794 patients with resected stage II-III esophageal or gastroesophageal junction cancer who had residual pathologic disease after neoadjuvant chemoradiotherapy to adjuvant nivolumab or placebo, regardless of PD-L1 expression [29]. Nivolumab approximately doubled median disease-free survival (22.4 versus 11.0 months; HR 0.69), establishing adjuvant checkpoint blockade as a new standard for patients without a pathologic complete response, with the benefit again not clearly gated by PD-L1 status [29]. On the neoadjuvant side, the ESCORT-NEO/NCCES01 trial randomized patients with resectable ESCC to neoadjuvant chemotherapy with or without camrelizumab followed by surgery, reporting improved pathologic complete response rates with the addition of immunotherapy [30]. Together, these perioperative data suggest that, unlike in the metastatic setting, PD-L1 expression may play a smaller role in guiding treatment decisions when immunotherapy is combined with curative-intent local therapy - though PD-L1-stratified survival data in the perioperative setting remain comparatively immature, and it remains unclear whether this apparent PD-L1 independence reflects true biological difference in the perioperative immune context or simply insufficient sample size to detect a subgroup effect analogous to that seen in the metastatic trials.

PD-L1 cutoff controversy: FDA reassessment and the 2024 ODAC vote

The apparent contradiction across trials - PD-L1-agnostic approvals in some settings (nivolumab, tislelizumab) alongside PD-L1-restricted approvals in others (pembrolizumab at CPS ≥10 in some lines) - together with the converging signal from the independent meta-analyses described above, prompted the FDA to commission its own pooled analysis across the CheckMate 648, KEYNOTE-590, and RATIONALE-306 first-line trials, focusing specifically on patients with PD-L1 expression below 1% [28,31]. This pooled analysis, comprising nearly 4,000 patients across the three trials, found no significant OS benefit from adding a PD-1 inhibitor to chemotherapy in the PD-L1-negative subgroup, with overlapping survival curves and a signal suggesting possible harm, in contrast to a clear and consistent benefit in patients with PD-L1 expression of 10% or higher across all three antibodies [28]. On September 26, 2024, the FDA's Oncologic Drugs Advisory Committee voted that the risk-benefit profile of first-line anti-PD-1 antibodies was not favorable in patients with metastatic or unresectable ESCC and PD-L1 expression below 1%, a vote that mirrored an equally decisive determination made the same day for PD-L1-negative gastric and gastroesophageal junction adenocarcinoma [28]. Committee members explicitly cited the near-complete overlap of survival curves below the 1% threshold, while acknowledging the statistical fragility inherent in subgroup analyses drawn from a relatively small number of PD-L1-negative patients across the three ESCC trials combined [28]. Following this recommendation, the FDA narrowed the labeled indications for nivolumab and pembrolizumab in ESCC, and in gastric/GEJ adenocarcinoma, to require PD-L1 expression of 1% or greater.

A subsequent retrospective analysis of RATIONALE-306 focusing specifically on patients with a PD-L1 TAP score ≥1% found that tislelizumab plus chemotherapy retained a favorable efficacy and safety profile in this biomarker-selected population, providing supportive data for a PD-L1-positive-restricted indication analogous to that adopted for nivolumab and pembrolizumab; the longer term follow-up of the same trial has continued to support this pattern [31,32]. Notably, however, exploratory analyses presented alongside these regulatory discussions, and echoed in the patient-level meta-analytic literature described above, suggested that the benefit-versus-toxicity calculus may not change abruptly at a PD-L1 expression of exactly 1%, but rather varies more continuously with expression level: pooled hazard ratios for OS were substantially better in patients with PD-L1 ≥10% than in those with PD-L1 in the 1%-10% range, even though both groups were nominally "PD-L1-positive" under a 1% threshold [20,28]. This gradation implies that a single cutoff, wherever it is set, will inevitably group together patients with a meaningfully different expected benefit, and it revives an argument several trial biostatisticians and pathologists have made publicly - that PD-L1 may be better conceptualized as a continuous predictor of the magnitude of benefit rather than a binary eligibility switch [20].

Assay heterogeneity as a confounder of the cutoff debate

A substantial part of the difficulty in fixing a single, generalizable PD-L1 threshold stems from the fact that the pivotal trials did not use a harmonized assay. CheckMate 648 used the PD-L1 IHC 28-8 pharmDx assay with tumor-cell scoring, KEYNOTE-590 used the 22C3 pharmDx assay with CPS, and RATIONALE-306 used the VENTANA SP263 assay with TAP scoring [2,3,7,8,13]. Although concordance studies show reasonable agreement between CPS and TAP at low cutoffs (overall percent agreement above 85% at a 1% threshold) [2,3], agreement is less robust at higher, clinically discriminating cutoffs such as CPS/TAP ≥10, and multicenter comparisons of different antibody clones in ESCC tissue reveal only moderate concordance with clone-dependent differences in sensitivity [4,5]. The 2026 patient-level meta-analysis discussed above made this point explicitly its central thesis, cautioning that cross-trial interpretation of PD-L1 thresholds in ESCC "requires caution" precisely because tumor proportion score, CPS, and TAP are related but non-identical constructs, generated on different platforms, and that treating a CPS ≥10 finding from one trial as informative for a TAP-based cutoff in another risks a category error [20]. During the ODAC proceedings, at least one pathologist testified that CPS is a highly subjective assay prone to producing different results on the same tissue block depending on the reader, and called for a single standardized PD-L1 test across drugs and indications - a recommendation the FDA acknowledged it has pursued unsuccessfully with manufacturers for years [28]. In practice, this means a patient's eligibility for first-line immunotherapy in ESCC may depend as much on which assay and antibody clone their pathology laboratory happens to use, and on whether tumoral versus stromal staining is weighted in the interpretation, as on their tumor's true biology - a problem that a single numeric cutoff cannot solve on its own [6,20].

Beyond PD-L1: emerging predictive biomarkers

Given the imperfect and assay-dependent nature of PD-L1 scoring, several groups have explored complementary biomarkers in ESCC. Exploratory genomic analyses from JUPITER-06 proposed a copy-number-alteration-corrected tumor mutational burden (ccTMB) and an esophageal cancer genome-based immuno-oncology classification (EGIC) scheme as predictors of long-term benefit from toripalimab plus chemotherapy, identifying alterations in the SWI/SNF chromatin-remodeling complex as associated with improved outcomes from combination immunochemotherapy independent of PD-L1 status [14]. Authors of the ESCORT-1st final analysis likewise concluded that the predictive value of PD-L1 alone appears limited in ESCC, and argued for deeper characterization of the tumor microenvironment - particularly tumor cell-immune cell interactions - to better identify patients likely to respond to anti-PD-1 therapy [11]. The tumor-versus-stroma distinction highlighted earlier in this review similarly points toward compartment-resolved digital pathology, rather than a single aggregate score, as a plausible refinement of current biomarker practice [6]. These genomic and microenvironmental approaches remain investigational and are not yet incorporated into regulatory labeling or clinical guidelines, but they illustrate that the field is actively searching for biomarkers that might supplement or eventually replace a PD-L1 percentage as the sole gatekeeper for immunotherapy access.

Guideline discordance and real-world practice

The rapid succession of trial readouts, each with its own PD-L1 assay, cutoff, and geographic patient population, has left international guideline bodies somewhat out of step with one another and with evolving FDA labeling. Major oncology guidelines have historically recommended first-line checkpoint inhibitor-chemotherapy combinations for advanced ESCC broadly, without uniformly mandating a specific PD-L1 threshold, reflecting the PD-L1-agnostic approvals that nivolumab and tislelizumab initially received in several regions. The 2024 U.S. label narrowing for nivolumab and pembrolizumab to PD-L1 CPS ≥1 creates a period of transition in which U.S. prescribing practice, European guidance, and practice in China or Japan - where camrelizumab, sintilimab, and toripalimab are used under separate regulatory frameworks with their own PD-L1 stipulations - may diverge for a time. This divergence has practical consequences: a patient with PD-L1 CPS of 0 who would previously have received nivolumab-chemotherapy under a PD-L1-agnostic U.S. label may now be directed toward chemotherapy alone, while an analogous patient treated in a jurisdiction that has not yet adopted the revised threshold could still receive immunotherapy. Community oncologists and pathologists will need clear, updated guidance - ideally harmonized across regulatory agencies - regarding which assay-specific cutoffs apply to which drug, since applying a CPS ≥1 threshold derived from a 22C3-based trial to a TAP score generated with a different antibody clone is not, strictly speaking, a validated substitution, even where population-level concordance data are reassuring [2,3,20].

Clinical implications and future directions

For the practicing oncologist, the net effect of this evolving evidence is a more nuanced, and in some ways more restrictive, approach to first-line immunotherapy selection in advanced ESCC than that existing even two years ago. Whereas early approvals for nivolumab and tislelizumab were largely PD-L1-agnostic, current U.S. labeling for both nivolumab and pembrolizumab now requires PD-L1 expression of 1% or greater, with an even stronger case for benefit at expression levels of 10% or greater [7,8,28,31]. Clinicians should therefore expect biomarker testing to remain mandatory rather than optional before initiating first-line immunotherapy, understand that the specific assay and scoring method used by their institution's pathology laboratory can influence the reported result, and interpret PD-L1 results as an indicator of the probable magnitude of benefit rather than a strict binary determinant of whether any benefit exists. Because assay and cutoff choices differ from country to country and between agents even within the same country, international guideline bodies will need to grapple with harmonization, and pathology laboratories will need clearer guidance on which assay-scoring combinations are considered interchangeable for a given drug. Future trials would benefit from prospectively incorporating a standardized assay, reporting PD-L1 expression as a continuous rather than dichotomized variable, distinguishing tumoral from stromal expression rather than reporting a single blended score, and pairing PD-L1 with emerging genomic or microenvironmental biomarkers such as ccTMB or SWI/SNF-complex status to build multi-marker predictive models [6,14,20]. Prospectively designed, adequately powered patient-level meta-analyses - built, ideally, on harmonized assay data collected at the outset rather than reconstructed retrospectively - represent a pragmatic near-term path to resolving the cutoff question more definitively than any single additional phase 3 trial is likely to achieve alone. Until such tools mature, PD-L1 testing - imperfect, assay-dependent, and now more of a continuous risk-stratifier than a strict gate - remains the best available, and therefore still clinically relevant, biomarker for guiding first-line immunotherapy decisions in advanced ESCC.

## Conclusions

Immune checkpoint inhibitors have become integral to the management of advanced ESCC across first-line, second-line, and perioperative settings, with numerous phase 3 trials of nivolumab, pembrolizumab, camrelizumab, sintilimab, tislelizumab, and toripalimab each demonstrating survival benefit. PD-L1 expression, whether measured by CPS or the more efficient TAP score, has correlated with the magnitude of that benefit across nearly every trial and across an increasingly large and consistent body of independent meta-analytic literature, culminating in a 2024 FDA pooled analysis and subsequent ODAC vote that formalized this relationship by restricting first-line indications to PD-L1-positive disease. However, persistent assay heterogeneity, only moderate concordance between antibody clones and scoring systems, a biologically meaningful distinction between tumoral and stromal PD-L1 expression, and evidence that benefit varies continuously rather than abruptly across the expression spectrum all suggest that a single percentage cutoff remains an imperfect proxy for the biology it is meant to capture. PD-L1 cutoffs are therefore still clinically relevant - the data supporting a threshold around 1% are now corroborated across multiple independent trials, meta-analyses, and a formal regulatory review - but they should be understood and communicated to patients as an approximate, assay-dependent stratifier of expected benefit rather than a precise biological boundary, pending the validation of complementary genomic and immunologic biomarkers.
